# Automated and app-based activation of first responders for prehospital cardiac arrest: an analysis of 16.500 activations of the KATRETTER system in Berlin

**DOI:** 10.1186/s13049-023-01152-3

**Published:** 2023-12-20

**Authors:** C. Pommerenke, S. Poloczek, F. Breuer, J. Wolff, J. Dahmen

**Affiliations:** 1 Charité University Berlin, corporate member of Freie Universität Berlin and Humboldt-Universität zu Berlin, Berlin, Germany; 2 Chief Medical Director, Emergency Medical Services, Fire Department, Berlin, Germany; 3 Emergency Medical Services Director, Rhine-Berg-District, Office for Fire Protection and Emergency Medical Service, Bergisch Gladbach, Germany; 4 Department of Anesthesia, Intensive Care and Emergency Medicine, Military Hospital Berlin, Berlin, Germany; 5 Department of Medicine, Health Faculty, University Witten/Herdecke, Witten, Germany

**Keywords:** Out-of-hospital cardiac arrest (MeSH), Crowdsourcing, Community response, Community first responder, Smart technology, Dispatcher, Emergency medical services (MeSH), Bystander CPR

## Abstract

**Background:**

Bystander CPR is one of the main independent factors contributing to better survival after out-of-hospital cardiac arrest. Simultaneously, the rate of bystander CPR in Germany is below the European average. First responder applications (apps) contribute to reducing the time period without CPR (no-flow time) until professional help can arrive on-scene.

**Methods:**

The KATRETTER app was introduced in Berlin as one of the first apps in Europe which do not require any medical qualifications to register as a first responder. The activation of volunteer first responders for suspected cardiac arrest cases through the Berlin Emergency Medical Services integrated control center was evaluated based on data collected between 16 Oct 2020 and 16 Oct 2022. Our descriptive analysis includes the number of registered first responders, number of activations, the number and percentages of accepted activations, as well as all reports where first responders arrived at the scene.

**Results:**

As of 15 Oct 2022, a total of 10,102 first responders were registered in the state of Berlin. During this specified period, there were 16.505 activations of the system for suspected out-of-hospital cardiac arrest. In 38.4% of the accepted cases, first responders documented patient contact, and in 34.6% of cases with patient contact, CPR was performed. Only 2% of registered first responders did not have any medical qualifications.

**Conclusions:**

Smartphone-based first responder applications should not be understood as a means of alerting professional help, but rather like a digitally amplified “call for help” in the vicinity of an emergency location. A large number of first responders can be recruited within 24 months, without large-scale public relations work necessary. No qualifications were required to become a first responder, contributing to a low-threshold registration process with the effect of a more widespread distribution of the app and cost reduction during implementation.

## Background

The number of out-of-hospital cardiac arrests (OHCA) reported worldwide is steadily rising [1]. In Europe, the incidence of OHCA is between 67 and 170 per 100,000 residents, and EMS (Emergency Medical Services) personnel attempt or continue resuscitation in around 50–60% of cases [2]. According to the German Resuscitation Registry [3], the incidence for prehospital resuscitation in Germany was 60.4 per 100,000 residents in 2021. Survival after OHCA is significantly higher with bystander CPR (cardiopulmonary resuscitation) at 17% in comparison to 9.5% without bystander resuscitation, regardless of the cause of arrest [4]. In Germany, from 2007 to 2019, only 35.1% of all OHCAs had bystander CPR performed, with automated external defibrillator (AED) usage at merely 1.4% [4]. Extensive efforts, including education, first aid courses, telephone-guided CPR (T-CPR), and the use of community first responder alerting systems/apps, have significantly increased the rate of bystander CPR in Germany to 42.6% [3]. However, this number remains below the European average of 58% and far from maximum values of up to 83% [2, 5].

In 2021, EMS resources in the State of Berlin, Germany, attended to 446,149 medical assistance requests (11,817/100,000 population) out of a total of 1,095,932 emergency calls received at the integrated emergency services control centre. Medical assistance requests have been on the rise in recent years [6], consistent with similar trends observed in other regions across Germany [7, 8]. In order to identify an out-of-hospital cardiac arrest (OHCA) during an emergency call and distinguish it from other emergency situations, the Berlin Fire Department control center employs a quality-controlled and evidence-based standardized software system for emergency call taking. The primary goal is to rapidly detect life-threatening conditions such as cardiac arrests and take appropriate immediate action with utmost reliability [9]. In 2019, emergency medical services registered 2595 prehospital resuscitations and processed 6383 calls with the keyword “cardiac arrest” in Berlin [10].

In addition to systematically establishing telephone-guided bystander CPR and ensuring widespread availability of publicly accessible AEDs, the widespread use of community first responder apps (CFR apps) is a central and indispensable component for increasing the survival rate of OHCA. Such apps are also recommended by the Global Resuscitation Alliance (GRA), the American Heart Association (AHA), and the European Resuscitation Council (ERC) [11–13]. These systems have also been found to significantly reduce the time without medical attention, allowing life-saving immediate CPR to begin as soon as possible [14].

With an ageing population, an increasing burden of disease, growing numbers of single-person households leading to social isolation, and inadequate private support networks, emergency services across Europe and North America face rising call volumes [15]. This trend is mirrored in Berlin, resulting in inevitable negative consequences for OHCA response times.

Due to these considerations, our objective was to establish a reliable, easy-to-operate and cost-effective CFR system that would work in a metropolitan area. Our strategy entailed the development of an in-app registration procedure that is entirely automated, targeting the entire population in the sense of a crowdsourcing approach as inclusive as possible, with no medical qualification requirements to become a first responder. Since 2017, the Berlin EMS have been working with “Fraunhofer FOKUS” to develop KATRETTER, a community first responder app, as part of a research collaboration, taking into account a lack of resources for administrative staff and advertising opportunities. In 2020, the global pandemic posed an additional challenge.

This paper outlines the low-cost implementation process of the KATRETTER app, covering an area of 3.7 million people. Additionally, we present the analyzed data from the implementation period of the project.

## Methods

16,505 Data sets of the Berlin KATRETTER system were included in the analysis and evaluated retrospectively. For this purpose, anonymized data was processed, thus excluding any identification of individual patients or first responders.

Data was collected from the KATRETTER system in the period starting 16 Oct 2020 (since its introduction) up until 16 Oct 2022. The cumulative number of app installations (installation of the app on a smartphone), the number of registered first responders (first responders who installed the app and then registered via SMS), the number of OHCA cases, the number of activations, the numbers and percentages of accepted activations, as well as all cases in which first responders arrived at the scene, were analyzed.

The KATRETTER app is a CFR mobile app that is freely available for download on multiple platforms (iOS, Android) in Germany. The KATRETTER system utilizes mobile networks and geolocation technology embedded in each mobile phone device, exploiting the high prevalence of smartphones in the population.

**Fig. 1:**
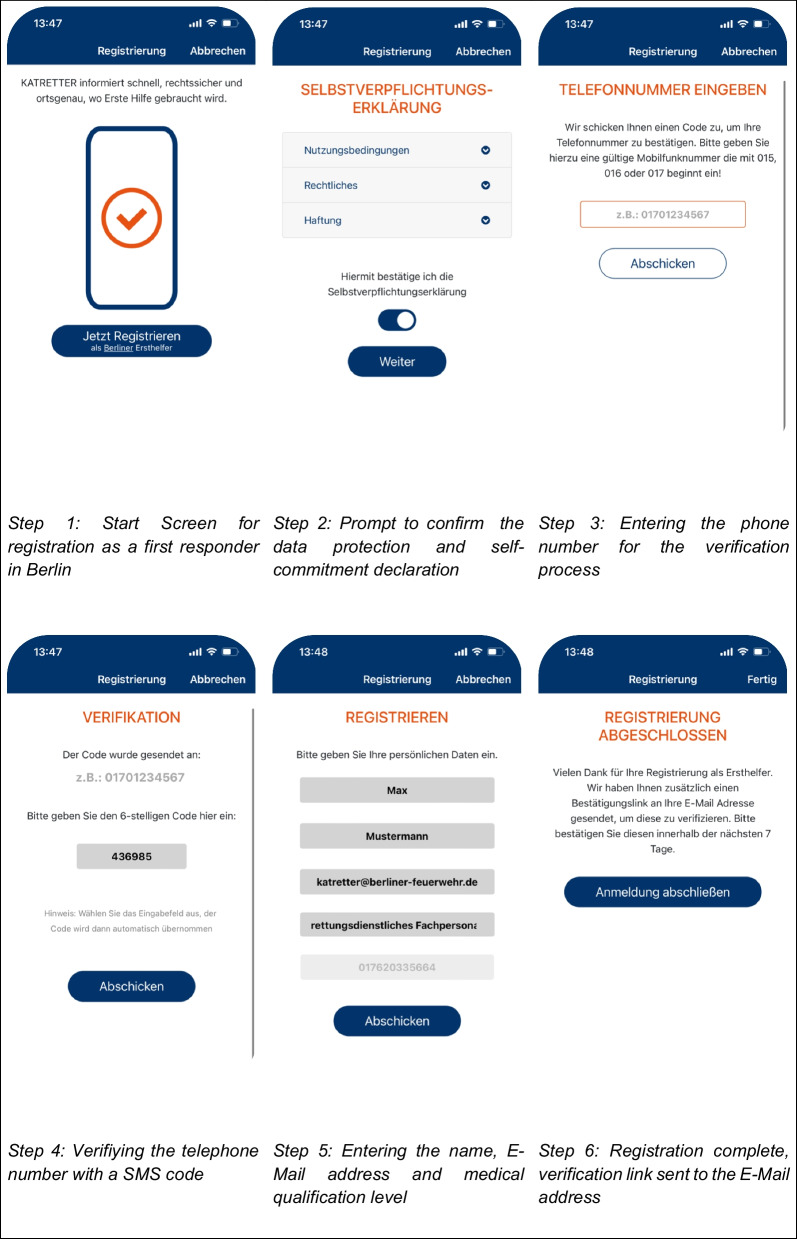
Registering as a new first-responder within the KATRETTER application in Berlin

In Berlin, a self-registration process is available for new first responders. A short, standardized questionnaire (Fig. 1) guides the new first responder, verifying their phone number through SMS and obtaining a privacy statement among other queries. The phone number validated using this method can later be used for contact. Once registration is complete, the first responder is not required to take any further administrative action and the new first responder can now be activated via the app on the verified mobile phone. This technology enables control centres to find volunteer first responders in the vicinity of an OHCA patient and activate them to perform basic life support (BLS) procedures.

The standardized medical emergency call handling system at the Berlin Control Centre enables seamless integration of the app into the dispatch process, requiring no additional effort on the part of emergency dispatchers. Following the guidelines of AHA and ERC, the standardized procedure for medical emergency calls first verifies the presence of consciousness and normal breathing. If both are absent, a dispatch code is assigned if OHCA is suspected, and the KATRETTER system automatically dispatches the nearest emergency resources and activates up to three first responders within a 500 m radius in the city centre and 1000 m in suburban areas. The distances and quantity of initial responders were established based on theoretical considerations and practical experience from the implementation phase. Furthermore, first responders are also activated in cases where individuals experience altered mental status following aspiration events, as a rapid decline into OHCA is anticipated under these circumstances. For every unconscious patient with questionable normal breathing, the emergency call takers count their breaths. First responders are subsequently activated via the KATRETTER system if deemed necessary. This method prevents the activation of first responders for every reported case of loss of consciousness—which leads to a higher rate of false-positives and unnecessary callouts—and was implemented during the observational period in February 2022 (see footnote in Table 1). This lead to a comparison of two phases in the results. OHCA in nursing homes and OHCA possibly caused by suicide or intoxication were also included. Activation does not occur for OHCAs presumed to be due to a traumatic cause (e.g. traffic accident) and for newborns younger than 30 days, due to different automatically generated dispatch codes through the software. This also applies to emergency calls where danger to the first responders cannot be ruled out (see Table 1).

**Table 1:** Automatically generated dispatch codes prompting first responder activation and similar excluded codes

	Code with suffix	Description
Automated first responder activation	09D01	Cardiac arrest—ineffective breathing
	09E01	Cardiac arrest
	09E02	Cardiac arrest—unknown if breathing
	11D02C	Altered mental status following aspiration event
	11E01 any suffix	Respiratory arrest after airway obstruction
	12D01/12D03	Cardiac arrest after seizure
	14E01 any suffix	Cardiac arrest after drowning accident, patient no longer in water
	23E01/23D01 any suffix	Cardiac arrest after overdose/poisoning
	31E01/31D01^a^	Cardiac arrest—ineffective/Agonal respiration
Excluded	03D01	Cardiac arrest after animal bite/attack
	04D01 any suffix	Cardiac arrest after bodily injury
	07D02 any suffix	Cardiac arrest after burn/scald
	09D02 any suffix	Probable dead person
	09E01	Cardiac arrest in newborn (< 30 days)
	09E02	Cardiac arrest—uncertain breathing in newborn (< 30 days)
	09E03	Cardiac arrest—recent hanging
	09E04	Cardiac arrest—strangulation
	09E05	Cardiac arrest—asphyxiation (due to external influence)
	14E02	Cardiac arrest after drowning accident, patient still under water
	15E01 any suffix	Cardiac arrest after electrical accident/lightning strike
	17D02 any suffix	Cardiac arrest after fall
	21D01 any suffix	Cardiac arrest with bleeding
	25D01 any suffix	Cardiac arrest in psychiatric emergency
	29D06	Cardiac arrest after traffic accident
	30D01	Cardiac arrest after injury
	34D03	Automatic accident report, person with respiratory arrest

^a^Because of a high number of false positive activations, we deleted the Code 31D02 during the observational period from the list in February 2022. This is the reason for the decrease of activations in Fig. 4

Upon accepting an alert in the KATRETTER app, first responders receive detailed information about the emergency location, including an in-app map, as well as the option to start navigation directly on the smartphone. However, the app currently lacks integration for alerting other first responders to retrieve the nearest AED. Upon arrival at the scene, first responders have the option to identify themselves via a digital badge in the app and send a timestamp to the control centre by clicking the “arrived” button. Once the mission is completed, it can be terminated within the app, and a brief digital questionnaire will appear for documentation and follow-up purposes. This instrument is used for the systematic psychosocial monitoring of first responders following their missions. If necessary, first responders can then be contacted via phone by Berlin Fire Department officials. The application also provides information on how to contact the control centre in case of an urgent need for (psychological) help.

The KATRETTER administration software is equipped with an export function, which we utilised to produce and export the dataset to Microsoft Excel. The export was initially carried out for ad hoc quality management analysis during the execution phase of the CFR system and app implementation. The extracted dataset was anonymised and did not contain any timestamps or location data. The datasets were derived from a simplified version of the commonly used MIND 3.1 dataset standard in the German rescue service. However, this more compact version only included information that is pertinent in the context of our analysis of the CFR system. The integrity of the data set was controlled using Microsoft Excel, which then transformed it into a format suitable for further descriptive analysis through the statistical software R.

The KATRETTER system is fully automated, so we had a complete data set without missing data. We didn’t use the time stamps for the arrival of the CFR because they are not reliable. The implementation process of KATRETTER was proved by the Berlin federal state commissioner for data protection. Every user of the app agree the data protection regulations in the installation process and get information about his rights belonging to the general data protections rules.

## Results

**Fig. 2:**
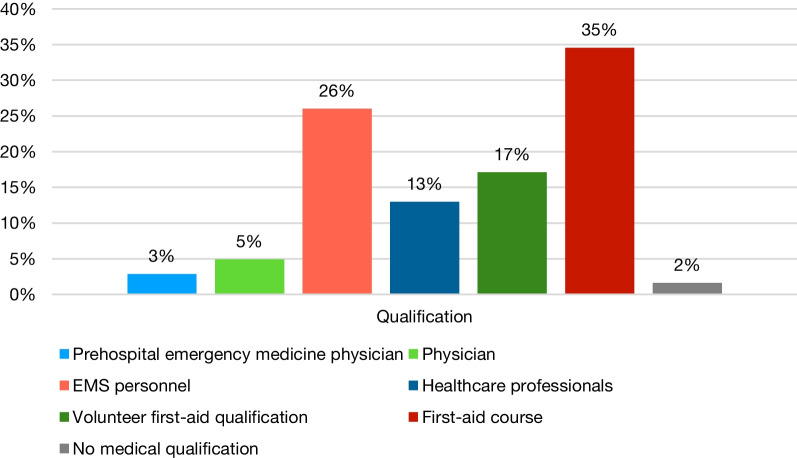
Qualifications of KATRETTER first-responders in the state of Berlin

Since its launch on 16th October 2020, the KATRETTER application has experienced a consistent rise in downloads, achieving over 100,000 app installations throughout Germany by 15th October 2022. Presently, 21 EMS control centres in Germany are linked to KATRETTER as of July 12th, 2023. [16] As of 15th October 2022, a sum of 10,102 initial responders have enrolled in the state of Berlin. In a region with approximately 3.7 million residents, the rate of first responders per 100,000 inhabitants is 274. As part of the registration process, first responders enter their medical qualifications as a requirement, as shown in Fig. 2.

**Fig. 3:**
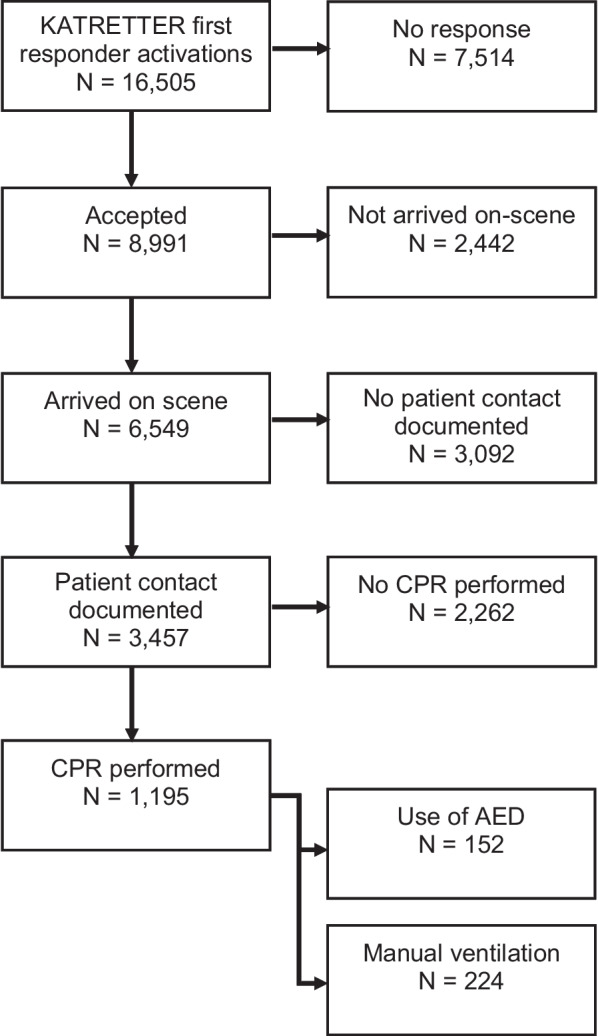
Overview of case numbers, AED: Automated external defibrillator

Between 16th October 2020 and 15th October 2022, there were 16,505 instances of KATRETTER being activated in Berlin in response to suspected Out-of-Hospital Cardiac Arrest (OHCA). The median monthly activation rate was 715 with a standard deviation (SD) of 183.4 and interquartile range (IQR) of 502–825. In 8991 cases, first responders accepted the app-based activation (median 384 per month, SD = 104.9, IQR = 274–440); in 39.7% (n = 6549) of suspected OHCA cases, at least one first responder arrived on scene (median 284 per month, SD = 80.8, IQR = 192–332). In 38.4% (n = 3457) of accepted cases, a patient contact was documented by the first responder (median 151 per month, SD = 44.5, IQR = 102–180). Of these, cardiopulmonary resuscitation was initiated or continued in 1.195 cases (median 48 per month, SD = 10.6, IQR = 45–54), representing 34.6% of the cases with patient contacts. No data was collected to calculate a quote of first responders arriving before EMS. An Automated External Defibrillator was used before EMS arrival in 12.7% (n = 152) of the resuscitation cases. The median usage per month was 7, with a standard deviation of 2.9 and an interquartile range of 4–8. Additionally, ventilation was noted as part of the resuscitation procedures in 18.7% (n = 224) of cases, with a median of 9 per month, an SD of 3.5, and an IQR of 6–12 (refer to Fig. 3).

**Fig. 4:**
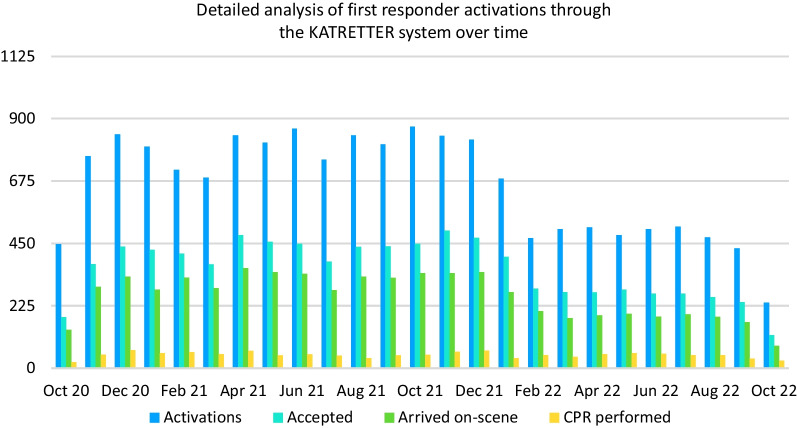
Detailed analysis of first responder activations through the KATRETTER system over time. *Note*: Because of a high number of false positive activations we deleted the Code 31D02 “Severe respiratory distress” from the list of dispatch codes which prompt automatic CFR activation in the dispatch system (see Table 1) on February 1st, 2022. This is the reason for the decrease of activations during that timeframe seen in this figure

During the study period, there was a decrease in the number of system activations by KATRETTER per month from a median of 810 (SD = 105.6, IQR = 743–840) in phase 1 to a median of 480 (SD = 86.2, IQR = 469–502) in phase 2. However, the number of resuscitations performed by first responders only exhibited a slight change from a median of 49.5 (SD = 11.4, IQR = 46–58) in phase 1 to a median of 47 (SD = 8.8, IQR = 41–51) per month in phase 2. Figure 4 shows these results.

**Fig. 5:**
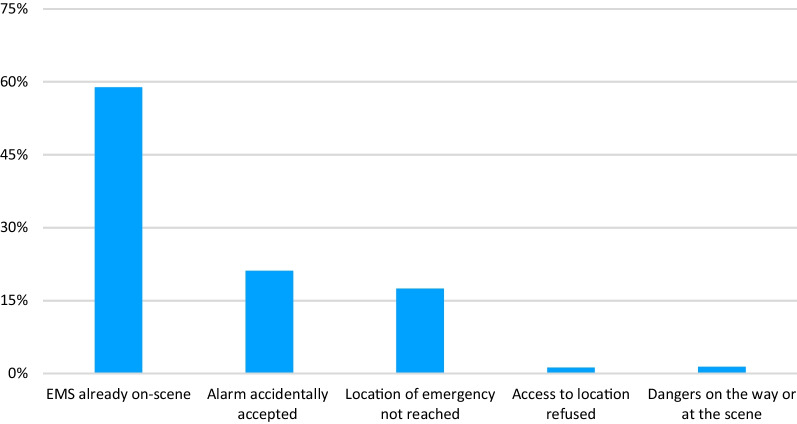
Distribution of causes for aborting a current mission

A total of 12,979 first responders accepted the activation. In 57.0% (n = 7403) of the cases examined, first responders terminated their mission without providing treatment. The most common reason, accounting for 58.9% (n = 4357) of these cases, was that the rescue service had already arrived at the scene. In 21.1% (n = 1564) of cases, first responders accepted the call by mistake. In 17.5% (n = 1294) of cases, first responders were unable to reach the scene. In 1.4% (n = 102) of cases, first responders were denied access to the scene/patient, and in 1.2% (n = 86) of cases, the reason for termination was documented as “danger on the way/at the scene” (see Fig. 5).

## Discussion

The application KATRETTER demonstrates for the first time the feasibility and advantages of automatic app-based first responder activation, crowdsourcing Basic Life Support treatment for OHCA patients in an entire German federal state. In line with the guidance of national and international medical bodies, it constitutes another indispensable element of crucial public health endeavors to enhance survival rates following out-of-hospital cardiac arrest (OHCA), in concert with telephone-guided CPR, regular CPR workshops in educational institutions and places of work, and the widespread use of publicly accessible AEDs [11, 13].

Smartphone-based first responder apps are in this context not to be viewed in a conventional sense as an alerting tool for professional help, but rather represent a form of digital call for help in the vicinity of the emergency location to bridge the time to EMS arrival with bystander CPR. Contrary, this very “call for help” was previously limited to sight or calling distance from the emergency scene [17]. The potential assistance from first responders mobilized through such applications is not restricted to executing basic life support measures. Instead, it could extend to tasks like directing emergency services to the crisis site, tending to family members, or providing the control centre with more comprehensive data on the situation, occurrences, and patient conditions. In this context, the question whether early bystander CPR or rather early defibrillation before arrival of EMS resources on scene will have the most impact on patient-centered outcomes remains to be answered. The results of a randomized-controlled trial done in Stockholm showed improved bystander CPR rates through activation of CFR. However, a survival benefit could not be demonstrated [18]. The same applies to a current study by Gregers et al. who, despite clearly showing increased rates of bystander CPR and defibrillation through an intensified CFR activation strategy, could not note improvements in return of spontaneous circulation or survival to hospital discharge. Study designs like these may often be under-powered to detect a change in secondary patient-centered outcomes like chance of survival [19]. A concept to especially boost early defibrillation rates by handing out ultraportable AED devices to CFR with the goal of earlier AED use in a higher number of OHCA activations is currently being studied in a trial in Australia (The First Responder Shock Trial; ACTRN12622000448741) with the primary outcome set to see a change in 30-day survival [20].

As for limitations, during the time period shortly after the first launch, cases were registered in which there was a premature termination during a mission due to a software error. Additionally, KATRETTER typically dispatches first responders to an OHCA only if the OHCA is also identified as such a (suspected) case during the emergency call. There may be instances where an OHCA is not identified despite the use of a standardized emergency call query during the emergency call taking process. Additionally, the cardiac arrest may have taken place after the emergency call had ended. Factors like the notification function of KATRETTER alarms being silenced or the geolocation function being turned off can impact compliance between the system and user. It is possible that there was an under-reporting of first responders arriving at the scene due to the absence of arrival timestamps sent by the first responder.

This analysis demonstrates that a significant quantity of first responders can be recruited within 24 months, without extensive advertising or publicity, and despite the pressures of a global pandemic. Nonetheless, as with other CFR systems such as Pulsepoint and myResponder, it is clear that the proportion of registered users accepting activations and responding is still relatively low [21, 22]. Significant contrasts can be observed between the registration process and requirements of the KATRETTER system in Berlin and other comparable applications across the globe [22]. In contrast, Pulsepoint in the USA generally restricts registration to professional helpers such as EMS personnel and healthcare professionals, while GoodSam in the UK mandates proof of a current BLS course. No special evidence is necessary to register with the HEARTRUNNER in Denmark or myResponder app in Singapore, which is equivalent to Berlin's KATRETTER system, allowing for registration through SMS validation on a low-threshold basis [21–25].

Other applications already implemented regionally in Germany, such as the “MOBILE RETTER”, “REGION LEBENSRETTER” or “CORHELPER” app, as well as previous implementation processes of the KATRETTER system in areas outside the state of Berlin, also require proof of qualification in a healthcare profession, and in some parts of Germany even additional specific induction and BLS courses [26–29].

Even in systems where individuals can register without proof of medical qualification, our data confirms that the vast majority of those who register possess some form of CPR qualification. Many of these individuals are medical professionals with regular experience in resuscitation. The benefits of a significantly reduced effort required by system operators in qualification management, as well as a lower threshold for qualified personnel to register, seem to outweigh other concerns.

Further functions distinguish the various nationally and internationally available apps, other than the registration process. For example, in addition to registering first responders, several apps can integrate other individuals or publicly accessible locations of an Automated External Defibrillator (AED), resulting in the simultaneous activation of a person to bring the nearest available AED to the scene of an out-of-hospital cardiac arrest (OHCA). Additional functions comprise the capability of initiating a video emergency call to the control centre or selecting in the app's first responder profile one's current mode of transport, including on foot, by bicycle or by car for calculating the optimal route and navigation [22, 23].

Recommendations for app-based cardiac first responder (CFR) activation systems in out-of-hospital cardiac arrest (OHCA) have been developed during a European consensus conference. This has exposed a considerable inconsistency in the use and design of these systems in Europe. It is generally agreed among the medical societies in Europe that country-wide systems should be implemented and consistently utilised. It is emphasised that bi-directional communication between first responders and control centres during and after activation for an OHCA is of particular importance [30]. This may be in line with observations from other CFR apps, some of which have identified a special psychosocial aftercare need for first responders after a mission, but further research is needed to confirm the extent of psychosocial aftercare demands [31, 32]. One of the foreseeable greatest challenges in Germany regarding the necessary nationwide implementation of CFR applications is, in addition to adequate funding, the possibility of connecting first responders across different control centers and different CFR apps throughout the country. Here, KATRETTER offers the prospect of better integration, connectivity, and cooperation through standardized, transparent interfaces, the ability to integrate first responders from other control center areas, and the fact that it has already been fully implemented in three German states.

Further, it is important to note that the invitation of citizens into first aid alerting systems presumably helps to strengthen individual medical emergency competency, but also more broadly speaking general health-related action ability and thus resilience of the population [33]*.*

## Conclusions

Smartphone-based first responder applications should not be understood as a means of alerting professional help. Rather, they should be viewed as a digitally amplified “call for help” in the vicinity of an emergency location, with a much better reach, focused on members of the public who are willing and capable to help at the time. This concept was a core idea during the launch phase of the KATRETTER system and has now been validated by the data sets analysed over the course of two years. In general, the expensive registration and user management costs for operators and users of other first-responder applications outside of Germany has arguably hindered the widespread implementation of app-based first-aid activation systems, as seen in other regions of Europe. Despite the availability of apps in isolated regions, the number of registered first-responders remains low in comparison to the population [27]]. By implementing a low-threshold registration process, widespread usage of the app and cost reduction could be attained. Additionally, the utilization of self-registration can greatly decrease operational efforts and integration costs for emergency service agencies.

## Data Availability

The datasets used and analyzed during the current study may be available from the corresponding author upon reasonable request.

## Abbreviations

AED: Automated external defibrillator
AHA: American Heart Association
App: Application
BLS: Basic life support
CFR app: Community first responder app
CPR: Cardiopulmonary resuscitation
EMS: Emergency Medical Services
ERC: European Resuscitation Council
GRA: Global resuscitation alliance
IQR: Interquartile range
OHCA: Out-of-hospital cardiac arrest
SD: Standard deviation
T-CPR: Telephone-guided CPR
